# *Withania somnifera* Improves Semen Quality in Stress-Related Male Fertility

**DOI:** 10.1093/ecam/nep138

**Published:** 2011-06-18

**Authors:** Abbas Ali Mahdi, Kamla Kant Shukla, Mohammad Kaleem Ahmad, Singh Rajender, Satya Narain Shankhwar, Vishwajeet Singh, Deepansh Dalela

**Affiliations:** ^1^Department of Biochemistry, C.S.M. Medical University, Lucknow 26003, India; ^2^Endrocrinology Division, Central Drug Research Institute, Lucknow 226001, India; ^3^Department of Urology, C.S.M. Medical University, Lucknow 226003, India

## Abstract

Stress has been reported to be a causative factor for male infertility. *Withania somnifera* has been documented in Ayurveda and Unani medicine system for its stress-combating properties. However, limited scientific literature is available on this aspect of *W. somnifera*. We undertook the present study to understand the role of stress in male infertility, and to test the ability of *W. somnifera* to combat stress and treat male infertility. We selected normozoospermic but infertile individuals (*N* = 60), further categorized in three groups: normozoospermic heavy smokers (*N* = 20), normozoospermics under psychological stress (*N* = 20) and normozoospermics with infertility of unknown etiology (*N* = 20). Normozoospermic fertile men (*N* = 60) were recruited as controls. The subjects were given root powder of *W. somnifera* at a rate of 5 g/day for 3 months. Measuring various biochemical and stress parameters before and after treatment, suggested a definite role of stress in male infertility and the ability of *W. somnifera* to treat stress-related infertility. Treatment resulted in a decrease in stress, improved the level of anti-oxidants and improved overall semen quality in a significant number of individuals. The treatment resulted in pregnancy in the partners of 14% of the patients.

## 1. Introduction

Infertility is defined as the inability of a couple to conceive after 12 months of unprotected regular sexual intercourse and it is estimated to affect 10%–15% of all couples. In almost half of such cases, a male factor is involved, but 15%–24% have unexplained etiology [1]. Most of the infertile men are reported to have a low sperm concentration and decreased motility as the cause. Alterations in spermatogenesis event may result in the release of immature or abnormal spermatozoa in the ejaculate. Although the total sperm count may still be within the normal fertile range these individuals may turn infertile due to large fraction of unfit spermatozoa [2]. Stress is one such potent factor to induce infertility in normozoospermic individuals. Defined by McGrady [3] more than half a century ago, the term “stress” has been used to include a variety of responses elicited by noxious or potentially noxious stimuli which may sensitize stress response. The hypothalamus-pituitary-adrenal (HPA) axis has been known to be involved in the stress response [4], and since HPA controls spermatogenesis, stress can be one possible contributor to the etiology of infertility [5].

The problem of infertility is closely related to psychological stress due to several other reasons as well. As a couple fails to achieve the expected goal of reproduction, the feelings of frustration and disappointment raise stress [6]. These feelings are compounded in couples experiencing infertility related disorders, requiring prolonged efforts to conceive. Several other factors, such as environmental pollutants, infections, occupational exposure to various chemicals, life- style changes and increased workload due to enormous competition at work place and economic recession accelerate psychological stress [7]. Cortisol has been designated as the stress hormone. There are reports that gonadal and sexual dysfunction are associated with elevated circulating cortisol levels [8]. Cortisol levels in circulation rise sharply in response to stress, resulting in testicular involution followed by significant drop in testosterone secretion [9]. There are reports that elevated psychological stress is associated with increased oxidant production and long-term exposure to stress factors may enhance the generation of reactive oxygen species (ROS). As spermatozoa contain considerable amount of lipids, they are more susceptible to oxidative stress which may initiate lipid peroxidation because of paucity of cytoplasmic enzymes responsible for scavenging ROS [10]. Cigarette smoking is known to be associated with subfertility in males and may result in decreased sperm concentration and low motility [11]. Chemical agents inhaled by smokers are known to affect male reproduction by directly affecting/inhibiting testicular function and spermatogenesis. Disturbance of the hypothalamus-pituitary-gonadal system or mild hypoxia caused by the disruption of the testicular microcirculation are possible explanations, but a direct toxic effect of the many chemical components in the cigarette smoke on the germinal epithelium is a more likely explanation. Chemical stress due to introduction of foreign chemicals in the body adversely affects metabolism. Overproduction of ROS, consequent to smoking, may be implicated as the main cause [12].

*Withania somnifera* has been documented in ancient Indian Ayurveda and Unani systems of medicine, for its capability to improve endurance against stress, general resistance against infections, retardation of the aging process and improvement of male sexual health in disorders such as psychogenic impotence and unexplained infertility. *W. somnifera* is a traditional medicine, the roots of which have been used not only as anti-stress agent but also as an aphrodisiac and male sexual stimulant [13, 14]. In the present study, we investigated the protective role of *W. somnifera* in infertile men who were either under psychological stress or were heavy smokers.

## 2. Methods

### 2.1. Chemicals

Nitroblue tetrazolium (NBT), thiobarbituric acid (TBA), phenazine methosulphate (PMS), nicotinamide adenine dinucleotide (NADH), 5,5′-dithio *bis* 2-nitrobenzoic acid (DTNB), 4,5 methyl thiazol-2-yl 2,5 diphenyltetrazolium bromide (MTT), nicotinamide adenine dinucleotide phosphate (NADPH) tricholoroacetic acid (TCA) and reduced glutathione (GSH) were purchased from Sigma Chemical Co., St. Louis, MO, USA. Radioimmunoassay kit for estimation of leuteinizing hormone (LH), follical stimulating hormone (FSH), Testosterone and prolactin (PRL) were obtained from Diagnostic Product Corporation, Los Angeles, CA, USA. All other reagents used were of high purity and analytical grade.

### 2.2. Plant Material

The roots of *W. somnifera* were purchased from an authorized dealer in Lucknow, India. They were identified and authenticated by Dr. MMAA Khan, Senior Lecturer, Department of Botany, Shia P.G. College, Lucknow, India, (Herbarium No. M-114 dated 19 December 2006). The roots were dried under shade and converted to a fine powder using a laboratory mill.

### 2.3. Study Protocol

The study protocol was approved by the Ethical Committee of the Chhatrapati Sahuji Maharaj Medical University (CSMMU), Lucknow. Before enrolment in the study, written informed consent from each subject was obtained in response to a fully written and verbal explanation of the nature of study. The potential participants, each with infertility persisting longer than a year, were clinically examined before being included in the study. Complete medical history of subjects and their female partners was also recorded. Subjects having diabetes, hypertension, arthritis, malignancy, tuberculosis, human immunodeficiency virus positive, infections, other endocrine disorders and those on drugs and conditions known to influence oxidative stress and serum cortisol level were excluded from this study.

### 2.4. Subjects and Sample Collection

A total of 121 men, aged between 25 and 38 years, were selected from the couples attending the Out Patient Department of Urology, C.S.M. Medical University, Lucknow. All subjects were instructed not to take any nutritional supplement or vitamins and not to change their dietary habits during the course of study. This study was conducted between January 2006 and 2008. The prospective study included four parallel groups of subjects: the control group comprised of 60 age-matched healthy men who had previously initiated at least one pregnancy and exhibited a normal semen profile (>20 × 10^6^ spermatozoa/ml; >40% motility and >40% normal morphology). Control subjects were not under stress, as evidenced by the study questionnaire and normal serum cortisol level.

The study group **c**omprised 60 subjects who were further allocated into three subgroups of 20 each on the basis of semen profiles. Normozoospermic infertile men had a normal semen profile (as defined for the control group above) and infertility of unknown etiology, and their female partners had undergone extensive infertility evaluation without showing a detectable gynecologic abnormality. These groups were (i) normozoospermic infertile men, (ii) normozoospermic infertile men under psychological stress and (iii) normozoospermic infertile men who were cigarette smokers. Among the smokers were individuals who had been smoking for the last ≥5 years, at the rate of ≥10 cigarettes per day. Psychological stress was assessed as per the protocol commonly known as State Anxiety Inventory [15]. All participants were asked to complete the questionnaire of the State Anxiety Inventory, as validated by Oner and Le Comple [16]. In this questionnaire, subjects were asked to describe how they feel “right now” by responding to 20 questions with a 4-point response format from “not at all” (score 1) to “extremely” (score 4). Total scores ranged from 20 to 80, with higher scores indicating greater anxiety. This measure has been shown to have high reliability and good contrast validity. Semen samples were collected from subjects after 3-4 days of sexual abstinence. Semen analyses were carried out according to the World Health Organization guidelines [17]. Venous blood samples were also withdrawn and serum separated for biochemical analysis.

### 2.5. Treatment

The infertile men were prescribed *W. somnifera* root powder, orally, in a single dose (5 g/day) for 3 months with a cup of skimmed milk [18]. Semen samples were collected twice, that is, first before administrating the medicine and second, after 3 months of treatment. For semen samples, morphological profiles were assessed within an hour of sample collection and biochemical profiles were evaluated within 2 days. During the course of study, the patients were monitored, on monthly basis, for liver function. The patients were followed for partner's pregnancy outcome for a period of 3 months after the treatment.

### 2.6. Preparation of Seminal Plasma

Semen samples were collected by masturbation after 4 days of abstinence into sterile plastic containers for analysis. The semen volume was recorded and an aliquot was taken to assess sperm motility. After liquefaction, semen samples were centrifuged at 1200 × g in cold (4°C) for 20 min for the separation of seminal plasma. The supernatant (seminal plasma) was centrifuged again at 10 000 × g in cold (4°C) for 30 min to eliminate all possible contaminating cells and stored at –20°C until analyzed. All biochemical estimations were carried out on seminal plasma.

### 2.7. Biochemical Assays on Seminal Plasma

Seminal plasma lipid peroxides (LPOs) were estimated according to the method of Ohkawa et al. [19], superoxide dismutase (SOD) and catalase activities according to McCord and Fridovich [20] and Aebi [21], respectively, and GSH and vitamin C according to Hissin and Hilf [22] and Gavella et al. [23], respectively. Vitamins A and E were measured by high-performance liquid chromatography (HPLC) as per the modified method of Omu et al. [24]. *α*-tocopherol acetate and retinol acetate were pipetted into an Eppendorf tube. To this, seminal plasma was added and vortex mixed. Hexane extract of vitamins A and E was aspirated out in a glass tube, dried under nitrogen stream and dissolved into methanol. Finally, this preparation was injected into HPLC apparatus fitted with a reverse phase C-18 stainless steel column. The vitamins were eluted with methanol at a flow rate of 1.5 ml/min for 15 min. The peak height and the curve area of vitamins A and E and their acetate were measured on ultraviolet detector with 292 mm filters to calculate the amount of these vitamins in seminal plasma.

### 2.8. Blood Biochemistry

Serum testosterone (T), LH, FSH and PRL were measured by a double antibody radioimmunoassay (RIA) method using Gamma Counter [25]. Intra and interassay coefficients of variation were 10.0, 14.0, 8.5 and 12.5%, respectively. Serum cortisol level of all the groups under study was assessed in the morning (8 a.m.) and evening (4 p.m.), before and after treatment according to the method of Foster and Dunn [26].

### 2.9. Statistical Analysis

Normal healthy fertile men of control group and infertile groups were compared using one-way ANOVA (analysis of variance) followed by Dunnett test. Infertile groups, before and after treatment, were compared with paired *t*-test. A *P* < .05 was considered statistically significant. The statistical analysis was performed on commercial software INSTAT 3.0 (Graph Pad Software, San Diego, CA).

## 3. Results

### 3.1. Semen Parameters

**Figure 1:**
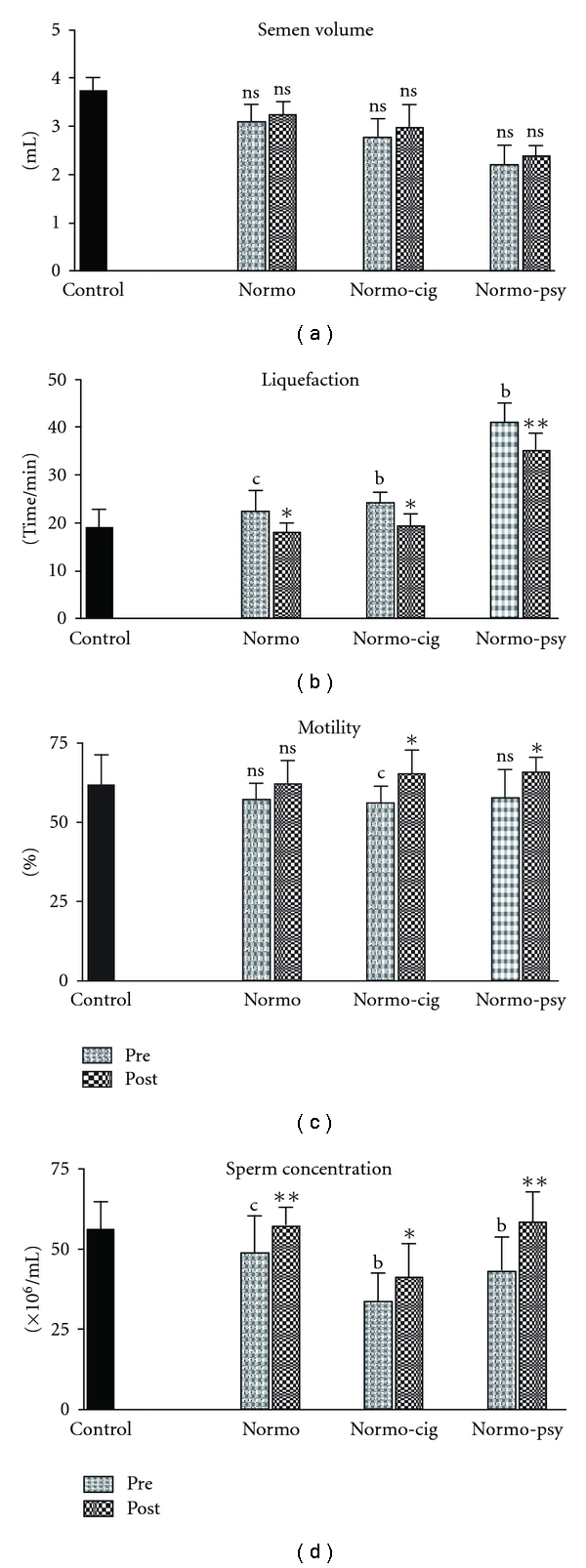
Semen profiles of “under stress” infertile men treated with *W. somnifera*. Pre: Pretreatment; Post: Post-treatment; Normo: normozoospermic; Normo cig: normozoospermic cigarette smokers; Normo psy: normozoospermic psychological stress. Each bar represents mean ± SD. Significance: ^b^ *P* < .01, ^c^ *P* < .05; compared with controls, **P < .01, *P < .05; compared with pretreatment subjects; NS, not significant.

General semen characteristics of different groups of subjects before and after treatment are presented in Figure 1. In normal healthy fertile men (control group), sperm concentration was 55.53 ± 9.06 × 10^6^/ml, motility was 60.18 ± 7.13% and liquefaction time of semen was 18.65 ± 4.05 min. Infertile groups exhibited decreased sperm concentration and/or slight decrease in motility. Semen liquefaction time was higher in infertile groups than control group. Treatment with *W. somnifera* significantly improved almost all the above semen parameters. Upon treatment the sperm concentration in normozoospermic men, cigarette smokers and those having psychological stress was increased by 17, 20 and 36%, respectively. Similarly motility of spermatozoa also increased by 9, 10 and 13% along with decrease in their semen liquefaction time by 19, 20 and 34%, as compared with the pretreatment parameters. Pregnancy outcome was 15% in normozoospermic men, 15% in men under psychological stress and 10% in cigarette smokers, giving an overall 14% rate of success.

### 3.2. Seminal Plasma Biochemistry

**Figure 2:**
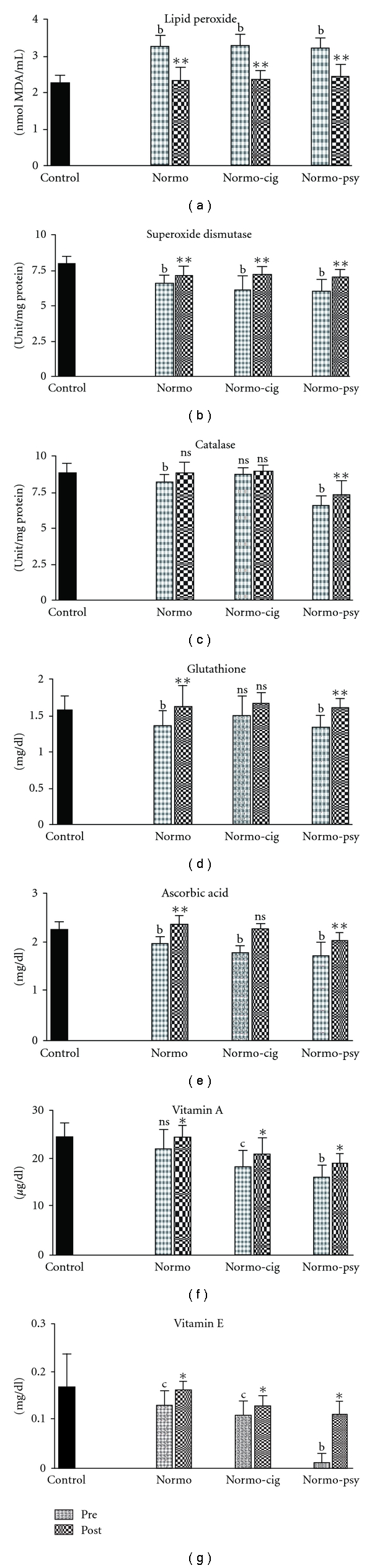
Biochemical parameters in patients treated with *W. somnifera*. Significance: ^b^ *P* < .01, ^c^ *P* < .05 (while comparing Pre with Control); **P < .01, *P < .05 (while comparing Post with Pre); NS: not significant (*P* > .05).

It was observed that the level of LPOs in seminal plasma of healthy fertile (control) men was 2.26 ± 0.25/nmol MDA/ml. However, seminal plasma levels of LPO in normozoospermic infertile men, normozoospermic cigarette smokers and normozoospermic men having psychological stress were elevated by 45, 44 and 40%, respectively. Following treatment with *W. somnifera* the levels of LPOs in seminal plasma of normozoospermic infertile men, normozoospermic cigarette smokers and normozoospermic men having psychological stress were decreased by 29, 27 and 23%, respectively, as compared with the pretreatment parameters (Figure 2).

We observed that SOD and catalase activity in seminal plasma of healthy fertile controls was 8.00 ± 0.51 unit/mg protein and 8.86 ± 0.71 unit/mg protein, respectively. These enzymes were found suppressed in infertile groups of normozoospermics, normozoospermic cigarette smokers and normozoospermics under psychological stress by 18, 23 and 24% for SOD, and 6, 2 and 26% for catalase, respectively. Treatment with *W. somnifera* restored SOD activity in seminal plasma of normozoospermic, normozoospermic cigarette smokers and normozoospermic psychological stress groups by 8, 18 and 17%, and catalase activity by 6, 3 and 11%, respectively, as compared with the pretreatment parameters (Figure 2). The levels of ascorbic acid and GSH in seminal plasma of control fertile group were 2.29 ± 0.14 mg/dl and 1.58 ± 0.21 mg/dl, respectively, and that in infertile normozoospermic males, cigarette smokers and males under psychological stress were low by 13, 22 and 25% for ascorbic acid and 13, 4 and 14% for GSH, respectively. Treatment with *W. somnifera* in normozoospermics, normozoospermic cigarette smokers and normozoospermics under psychological stress groups recovered ascorbic acid levels by 19, 25 and 18%, respectively, and also the levels of GSH by 19, 10, 20%, respectively, as compared with pretreatment parameters. Vitamins A and E were decreased in all groups. After treatment, their levels were increased in all subjects by a significant percentage.

### 3.3. Blood Biochemistry

#### 3.3.1. Hormones

**Figure 3:**
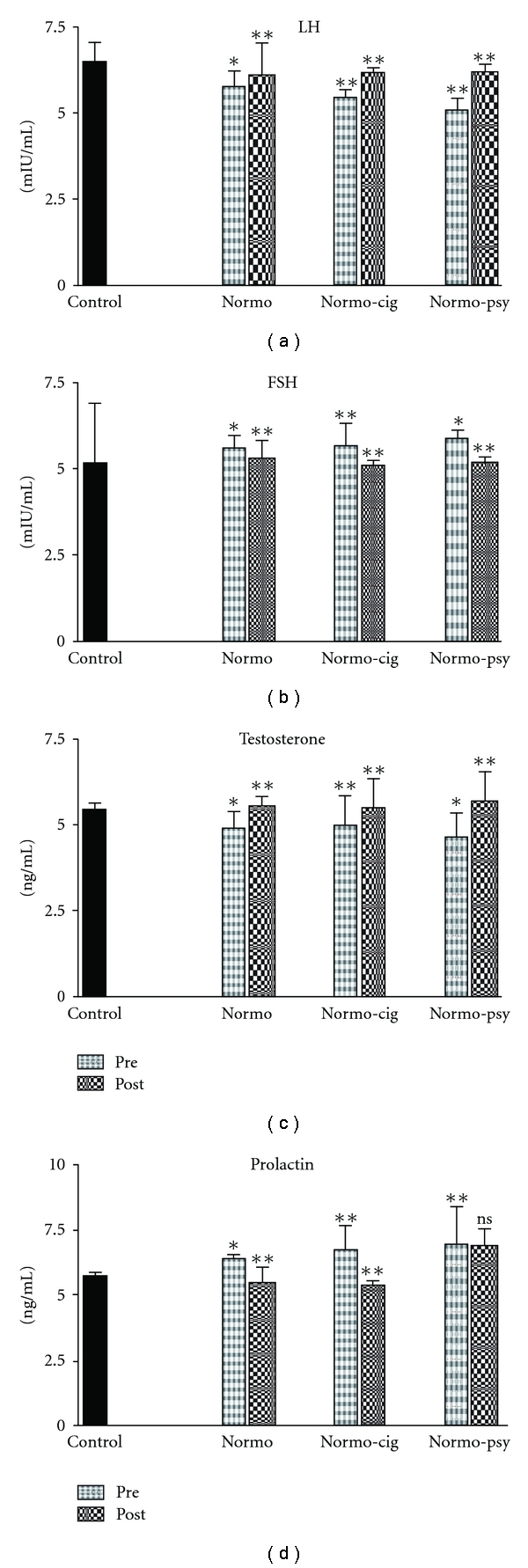
Bar diagram showing average hormonal levels in patients before and after treatment with *W. somnifera*. The asterisks on bars of pretreatment show significant difference from control and on post-treatment show significant difference from pretreatment. The error bars show standard deviation.

Serum testosterone level in control group was 5.41 ± 0.21 ng/ml, these levels were decreased in normozoospermic infertile group by 10%, normozoospermic cigarette smokers group by 8% and normozoospermic psychological stress group by 14% in comparison with control group (Figure 3). After treatment with *W. somnifera*, testosterone level improved in normozoospermics by 13%, normozoospermic cigarette smokers by 10% and infertile normozoospermics under psychological stress by 22%. Similarly, compared with the control group, serum LH level was low in normozoospermic infertile group by 11%, normozoospermic cigarette smokers group by 16% and normozoospermic psychological stress group by 21%. Treatment with *W. somnifera* recovered the level of LH in these groups by 5, 14 and 22, respectively. Among normozoospermic group, normozoospermic cigarette smokers group and normozoospermic psychological stress group FSH was raised by 8, 10, 13% and PRL by 12, 17 and 20%, respectively. After treatment with *W. somnifera*, these levels were significantly reduced. Intra- and inter-assay coefficients of variation in testosterone, LH, FSH and PRL were 10.5, 12.6, 9.5 and 12.5%, respectively.

#### 3.3.2. Cortisol

**Figure 4:**
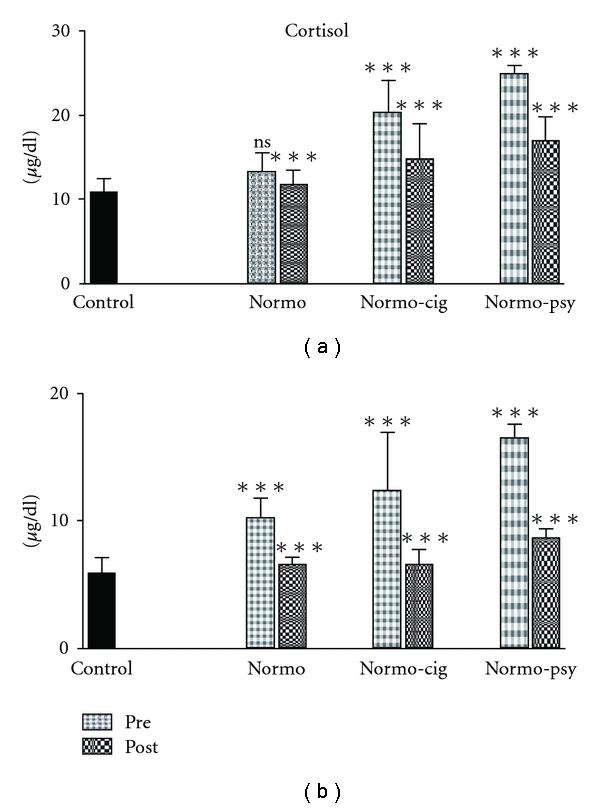
Bar diagram showing average serum cortisol levels at 8 a.m. (a) and 4 p.m. (b) in patients before and after treatment with *W. somnifera*. The asterisks on bars of pretreatment group show significant difference from control and posttreatment group show significant difference from pretreatment. The error bars show standard deviation.

The mean serum cortisol level of healthy fertile men in the morning was 10.84 ± 1.63 *μ*g/dl (Figure 4). However, these levels in normozoospermic, normozoospermic cigarette smokers and normozoospermic psychological stress groups were found elevated by 22, 88 and 129%, respectively. After treatment with *W. somnifera*, cortisol levels of normozoospermic, normozoospermic cigarette smokers and normozoospermic psychological stress were found significantly decreased by 11, 28 and 32%, respectively. Similarly the serum cortisol level observed at 4 p.m. in the control group was 5.8 ± 1.33 *μ*g/dl. However, these levels were increased in normozoospermic, normozoospermic cigarette smokers and normozoospermic psychological stress groups. Cortisol reading at 4 p.m. was also found significantly decreased in treated men by 36, 48 and 48%, respectively, as compared with pretreatment parameters.

## 4. Discussion

Normozoospermic infertile men are unable to fertilize a woman despite sperm concentration in the range considered normal. Therefore, poor overall semen quality might be responsible for infertility in such individuals. Our results pertaining to treatment of normozoospermic infertile men with *W. somnifera*, who were under psychological stress or were cigarette smokers, demonstrate improvement of their sperm count and motility significantly, along with reduction of stress and serum cortisol levels [27]. There are reports showing that *W. somnifera* possesses antioxidant, adaptogenic and aphrodisiac activities apart from having some neurotransmitters [28]. Our study confirms these properties, along with demonstrating the capability of this herb to improve male factor fertility in idiopathic cases.

This study offers useful information in at least three areas. First, it confirms the role of stress in male infertility. The catecholamines and serotonin are biogenic amines that are produced under stress and may have direct effect on the hypothalamus promoting the release of hypothalamic-releasing hormones. Epinephrine and norepinephrine released from the adrenal medulla may also affect the testis by changing local blood flow, as these hormones are known to produce vasoconstriction in other target tissues. Psychological stress may affect brain function and biological clock resulting in disturbance of hypothalamo-pituitary control of hormone production. The effect on the brain is ultimately manifested in other organs owing to hormonal regulation through hypothalamo-pituitary axis. This disturbance in hypothalamus-pituitary-gonadal axis may adversely affect spermatogenesis [29]. There have been several reports linking male infertility to stress [5]. Cortisol is the hormone produced in response to stress [8], and increased level of cortisol may reduce the functional activity of LH, thereby reducing testosterone level [30]. Psychological stress leads to low testosterone levels due to reduction in LH pulse frequency. Reduced testosterone level in turn reduces libido and leads to oligospermatogenesis [31]. The reduction of stress level and improvement in male factor fertility as a result of treatment with *W. somnifera*, offer direct evidence in favor of stress as a cause of male infertility.

Second, life style has a great deal to do with overall mental and general health. Life style factors such as smoking have been suggested to contribute to a number of diseases including male infertility. It has been reported that tobacco smoke contains some of the most deadly toxic chemicals. Smokers inhale directly and absorb the following substances: nicotine, carbon monoxide, nitrogen oxide, mutagenic pyrolysis-derived compounds and cadmium [32]. Most of them are known to be mutagens, aneugens or carcinogens, directly affecting male and female gametes and embryos. Additionally, there is a correlation between cigarette smoking in infertile men and increased leukocyte infiltration into semen [33]. The latter has been linked to significantly increased levels of seminal ROS. Under compromised conditions, such a stress may adversely affect reproductive health leading to infertility.

Third, *W. somnifera* has the capability of combating stress-induced infertility. The effect was obvious given a significant number (14%) of pregnancy outcome (15% in normozoospermic men, 15% in men under psychological stress and 10% in cigarette smokers) upon treatment. This activity may be due to the presence of a number of alkaloids, ergostane steroids and amino acids, including tryptophan, central nervous system inhibitors, centrally acting hypotensive agents, GABA agonists and serotonin agonists in the roots of this herb [34]. There are reports that *W. somnifera* is rich in neurotransmitters, besides having several other alkaloids and flavonoids [35]. It has been earlier reported that the flavonoids of *W. somnifera* possess potent antioxidant activity and treatment with *W. somnifera* may counteract the formation of ROS in infertile men [36]. The active principles of *W. somnifera*, sitoindosides VII-X and withaferin A (glycowithanolides), have been shown to reactivate the major free radical scavenging enzymes *in vivo* [37]. Treatment with *W. somnifera* in our study presented a direct evidence for its anti-stress properties. The extent of effect of the treatment was dependent on the percentage deviation of the parameter from the normal values, indicating adaptogenic nature of *W. somnifera*. For example, a parameter showing more deviation from the normal value in a particular group pretreatment, showed more percent change post-treatment (Figure 4). Nearly all the parameters were most severely deviated in men having psychological stress followed by cigarette smokers and normozoospermic individuals, and showed similar trend in percent change with minimum change in normozoospermic individuals. This was true for all parameters except PRL in men under the psychological stress group where the percent change upon treatment was only little and could not be explained.

**Figure 5:**
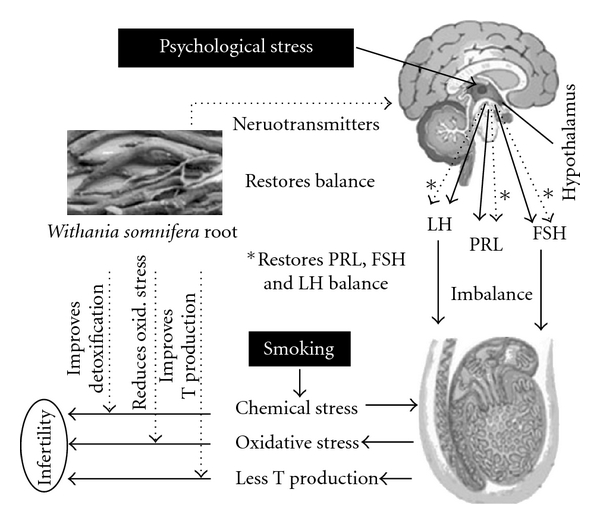
The proposed mechanism of action of *W. somnifera*. The solid lines in the figure indicate the situation in infertile subjects where psychological stress and smoking may contribute to infertility. The dotted lines indicate the points of action of *W. somnifera*, which acts by counteracting the stressors.

As discussed above, infertility in psychological stress group could be attributed to a variety of factors responsible for inducing psychological stress. In smoking group, it could be attributed to long-term exposure to xenobiotic compounds and associated health hazards. However, it remains interesting to explore the cause(s) of infertility in normozoospermic infertile group. Irrespective of the cause(s), the treatment offered by *W. somnifera* in the three groups indicates the presence of diverse active constituents in this herb. Though the exact mechanism of action of this herb needs further exploration of individual constituents, on the basis of above observations, we have proposed its possible mechanism of action (Figure 5). Among the major effects, it balances hormone levels, reduces oxidative stress and possibly improves detoxification processes in the body. We have earlier shown that disturbed hormone levels correlate well with infertility [5]; therefore, correction of this imbalance by *W. somnifera* could be one of the major factors contributing to fertility improvement. It could be a combination of direct and indirect effects of this herb to combat stress by pleuripotent effecter constituents. It would be worthwhile to stress that such an effect is more likely in its natural mixture form than isolating one or more individual fractions that may not yield similar effects. However, it is still a good idea to fractionate this herb to understand its mechanism of action.

## Funding

Grant 5/10/8/2004-RHN from the Indian Council of Medical Research, New Delhi, India.

## References

[1] Sikka SC (2001). Relative impact of oxidative stress on male reproductive function. *Current Medicinal Chemistry*.

[2] Mahdi AA, Bano F, Singh R, Singh RK, Siddiqui MS, Hasan M (1999). Seminal plasma superoxide dismutase and catalase activities in infertile men. *Journal of Medical Sciences Research*.

[3] McGrady AV (1984). Effects of psychological stress on male reproduction: a review. *Archives of Andrology*.

[4] Sikka SC, Wang R (2008). Endocrine disruptors and estrogenic effects on male reproductive axis. *Asian Journal of Andrology*.

[5] Shukla KK, Mahdi AA, Ahmad MK, Shankhwar SN, Rajender S, Jaiswar SP (2009). *Mucuna pruriens* improves male fertility by its action on the hypothalamus-pituitary-gonadal axis. *Fertility and Sterility*.

[6] Hardy MP, Gao H-B, Dong Q (2005). Stress hormone and male reproductive function. *Cell and Tissue Research*.

[7] Nagro-Vilar A (1993). Stress and other environmental factors affecting fertility in men and women; overview. *Environmental Health Perspectives*.

[8] Cowen PJ (2002). Cortisol, serotonin and depression: all stressed out?. *British Journal of Psychiatry*.

[9] Shukla KK, Mahdi AA, Shankwar SN, Ahmad MK (2008). Effect of *Mucuna pruriens* on hormonal status and semen quality in infertile males. *Contraception*.

[10] Bano F, Singh RK, Singh R, Siddiqui MS, Mahdi AA (1999). Seminal plasma lipid peroxide levels in infertile men. *Journal of Endocrinology and Reproduction*.

[11] Ramlau-Hansen CH, Thulstrup AM, Aggerholm AS, Jensen MS, Toft G, Bonde JP (2007). Is smoking a risk factor for decreased semen quality? A cross-sectional analysis. *Human Reproduction*.

[12] Soares SR, Melo MA (2008). Cigarette smoking and reproductive function. *Current Opinion in Obstetrics and Gynecology*.

[13] Shukla KK, Mahdi AA, Ahmad MK, Shankwar SN, Jaiswar SP, Tiwari SC ( 2010). *Mucuna pruriens* reduces stress and improves the quality of semen in infertile males. *Evidence-Based Complementary and Alternative Medicine*.

[14] Ahmad MK, Mahdi AA, Shukla KK, Islam N, Jaiswar SP, Ahmad S (2008). Effect of *Mucuna pruriens* on semen profile and biochemical parameters in seminal plasma of infertile men. *Fertility and Sterility*.

[15] Spielberger CD, Gorsuch RL, Lushene RE (1970). *STAI Manual for the State-Trait Anxiety Inventory*.

[16] Oner N, Le Comple A (1998). *DurumLuluk-Surekilik Kaygi Envanteri: Ei Kitabi*.

[17] World Health Organization (1999). *Laboratory Manual for The Examination oF Human Semen and Sperm Cervical Mucus Interaction*.

[18] Singh D, Singh D (1974). Konch (Kiwach). *Unani Dravvyagunadarsh*.

[19] Ohkawa H, Ohishi N, Yagi K (1979). Assay for lipid peroxides in animal tissues by thiobarbituric acid reaction. *Analytical Biochemistry*.

[20] McCord JM, Fridovich I (1969). Superoxide dismutase. An enzymic function for erythrocuprein (hemocuprein). *Journal of Biological Chemistry*.

[21] Aebi H, Bergmeyer HU (1974). Catalase. *Methods of Enzymatic Analysis*.

[22] Hissin PJ, Hilf R (1976). A fluorometric method for determination of oxidized and reduced glutathione in tissues. *Analytical Biochemistry*.

[23] Gavella M, Lipovac V, Vučić M, Ročić B (1997). Evaluation of ascorbate and urate antioxidant capacity in human semen. *Andrologia*.

[24] Omu AE, Fatinikun T, Mannazhath N, Abraham S (1999). Significance of simultaneous determination of serum and seminal plasma *α*-tocopherol and retinol in infertile men by high-performance liquid chromatography. *Andrologia*.

[25] Midgley AR (1967). Radioimmunoassay for human follicle-stimulating hormone. *Journal of Clinical Endocrinology and Metabolism*.

[26] Foster LB, Dunn RT (1974). Single antibody technique for radioimmunoassay of cortisol in unextracted serum or plasma. *Clinical Chemistry*.

[27] Bhat MS, Rao G, Murthy KD, Bhat PG (2007). Housing in pyramid counteracts neuroendocrine and oxidative stress caused by chronic restraint in rats. *Evidence-Based Complementary and Alternative Medicine*.

[28] Sharma S, Dhanukar S, Karandikar SM (1985). Effect of long term administration of the roots of *Ashwaganda* and *Shatawari*. *Drugs*.

[29] Jensen MS, Mabeck LM, Toft G, Thulstrup AM, Bonde JP (2005). Lower sperm counts following prenatal tobacco exposure. *Human Reproduction*.

[30] Llayperuma I, Ratnasooriya WD, Weerasooria TR (2004). Effect of *Withania somnifera* root extract on the sexual behavior of male rats. *Asian Journal of Andrology*.

[31] Merino G, Carranza Lira S, Martínez-Chéquer JC (1998). Effects of cigarette smoking on semen characteristics of a population in Mexico. *Archives of Andrology*.

[32] Sangwan RS, Das Chaurasiya N, Lal P (2007). Withanolide A biogeneration in in vitro shoot cultures of *Ashwagandha* (Withania somnifera Dunal), a main medicinal plant in ayurveda. *Chemical and Pharmaceutical Bulletin*.

[33] Pook M, Krause W (2005). Stress reduction in male infertility patients: a randomized, controlled trial. *Fertility and Sterility*.

[34] Sepaniak S, Forges T, Gerard H, Foliguet B, Bene M-C, Monnier-Barbarino P (2006). The influence of cigarette smoking on human sperm quality and DNA fragmentation. *Toxicology*.

[35] Smith CG (1982). Drug effects on male sexual function. *Clinical Obstetrics and Gynecology*.

[36] Arambewela L, Silva R (1999). *Withania Somnifera*.

[37] Agmo A, Paredes RG, Sierra L, Garces I (1997). The inhibitory effects on sexual behaviour and ambulatory activity of the mixed GA agonist proabide are differentially blocked by GABA receptor agonists. *Psychopharmacology*.

